# Complete genome sequence of *“Enterobacter lignolyticus” SCF1*

**DOI:** 10.4056/sigs.2104875

**Published:** 2011-09-23

**Authors:** Kristen M. DeAngelis, Patrik D’Haeseleer, Dylan Chivian, Julian L. Fortney, Jane Khudyakov, Blake Simmons, Hannah Woo, Adam P. Arkin, Karen Walston Davenport, Lynne Goodwin, Amy Chen, Natalia Ivanova, Nikos C. Kyrpides, Konstantinos Mavromatis, Tanja Woyke, Terry C. Hazen

**Affiliations:** 1 Ecology Department, Lawrence Berkeley National Laboratory, Berkeley CA USA; 2 Microbial Communities Group, Deconstruction Division, Joint BioEnergy Institute, Emeryville CA USA; 3 Lawrence Livermore National Laboratory, Livermore CA USA; 4 Physical Biosciences Division, Lawrence Berkeley National Laboratory, Berkeley CA USA; 5 Technologies Division, Joint BioEnergy Institute, Emeryville CA USA; 6 Sandia National Lab, Livermore CA USA; 7 Los Alamos National Laboratory, Los Alamos NM USA; 8 Department of Energy Joint Genome Institute, Walnut Creek CA USA

**Keywords:** Anaerobic lignin degradation, tropical forest soil isolate, facultative anaerobe

## Abstract

In an effort to discover anaerobic bacteria capable of lignin degradation, we isolated *“Enterobacter lignolyticus”* SCF1 on minimal media with alkali lignin as the sole source of carbon. This organism was isolated anaerobically from tropical forest soils collected from the Short Cloud Forest site in the El Yunque National Forest in Puerto Rico, USA, part of the Luquillo Long-Term Ecological Research Station. At this site, the soils experience strong fluctuations in redox potential and are net methane producers. Because of its ability to grow on lignin anaerobically, we sequenced the genome. The genome of *“E. lignolyticus”* SCF1 is 4.81 Mbp with no detected plasmids, and includes a relatively small arsenal of lignocellulolytic carbohydrate active enzymes. Lignin degradation was observed in culture, and the genome revealed two putative laccases, a putative peroxidase, and a complete 4-hydroxyphenylacetate degradation pathway encoded in a single gene cluster.

## Introduction

One of the biggest barriers to efficient lignocellulose deconstruction is the problem of lignin, both occluding the action of cellulases and as wasteful lignin by-products. Tropical forest soils are the sites of very high rates of decomposition, accompanied by very low and fluctuating redox potential conditions [1,2]. Because early stage decomposition is typically dominated by fungi and the free-radical generating oxidative enzymes phenol oxidase and peroxidase [3,4], we targeted anaerobic tropical forest soils with the idea that they would be dominated by bacterial rather than fungal decomposers. To discover organisms that were capable of breaking down lignin without the use of oxygen free radicals, we isolated *“Enterobacter lignolyticus”* SCF1 under anaerobic conditions using lignin as the sole carbon source. In addition to this, it has been observed to withstand high concentrations of ionic liquids [5], and thus was targeted for whole genome sequencing.

## Organism information

*“E. lignolyticus”* SCF1 was isolated from soil collected from the Short Cloud Forest site in the El Yunque experimental forest, part of the Luquillo Long-Term Ecological Research Station in Luquillo, Puerto Rico, USA (Table 1). Soils were diluted in water and inoculated into roll tubes containing MOD-CCMA media with alkali lignin as the source of carbon. MOD-CCMA media consists of 2.8 g L^-1^ NaCl, 0.1 g L^-1^ KCl, 27 mM MgCl_2_, 1 mM CaCl_2_, 1.25 mM NH_4_Cl, 9.76 g L^-1^ MES, 1.1 ml L^-1^ K_2_HPO_4_, 12.5 ml L^-1^ trace minerals [19,20], and 1 ml L^-1^ Thauer’s vitamins [21]. Tubes were incubated at room temperature for up to 12 weeks, at which point the colony was picked, grown in 10% tryptic soy broth (TSB), and characterized.

**Table 1:** Classification and general features of *“Enterobacter lignolyticus”* SCF1

**MIGS ID**	**Property**	**Term**	**Evidence code**
	Current classification	Domain *Bacteria*	TAS[6]
		Phylum *Proteobacteria*	TAS[7]
		Class *Gammaproteobacteria*	TAS[8,9]
		Order *Enterobacteriales*	TAS[10]
		Family *Enterobacteriaceae*	TAS[11-13]
		Genus *Enterobacter*	TAS[11,13-16]
		Species *“Enterobacter lignolyticus”*	
		Strain SCF	
	Gram stain	negative	NAS
	Cell shape	rod	IDA
	Motility	motile via flagella	IDA
	Sporulation	non-sporulating	IDA
	Temperature range	Mesophile	
	Optimum temperature	30°C	
	Carbon source	glucose, xylose, others; see Table 8	IDA
	Energy source		
	Terminal electron receptor		
MIGS-6	Habitat	Soil collected from a subtropical lower montane wet forest	TAS [17]
MIGS-6.3	Salinity	Can tolerate up to 0.75 M NaCl, 1 M KCl, 0.3 M NaOAc, 0.3 M KOAc. Growth in 10% trypticase soy broth is improved with 0.125 M NaCl	TAS [5]
MIGS-22	Oxygen	facultative aerobe; grows well under completely oxic and anoxic conditions	IDA
MIGS-15	Biotic relationship	free-living	IDA
MIGS-14	Pathogenicity	no	
MIGS-4	Geographic location	Luquillo Experimental Forest, Puerto Rico	IDA
MIGS-5	Sample collection time	July 2009	IDA
MIGS-4.1	Latitude	18.268N	IDA
MIGS-4.2	Longitude	65.760 W	IDA
MIGS-4.3	Depth	10 cm	IDA
MIGS-4.4	Altitude	1027 msl	IDA
			

a) Evidence codes - IDA: Inferred from Direct Assay; TAS: Traceable Author Statement (i.e., a direct report exists in the literature); NAS: Non-traceable Author Statement (i.e., not directly observed for the living, isolated sample, but based on a generally accepted property for the species, or anecdotal evidence). These evidence codes are from the Gene Ontology project [18].

When grown on 10% TSB agar plates, SCF1 colonies are translucent white, slightly irregular in shape with wavy margins, and have a shiny smooth surface. SCF1 was determined to be a non-sporulating strain based on a Pasteurization test. To do this, a suspension of SCF1 cells was heated at 80°C for 10 minutes. 5μl of heated culture and non-heated control culture were both spotted onto 10% TSB agar and incubated for growth for 3 days at room temperature. The non-heated cells grew while the heated culture did not, indicating the absence of heat-resistant spores.

**Figure 1:**
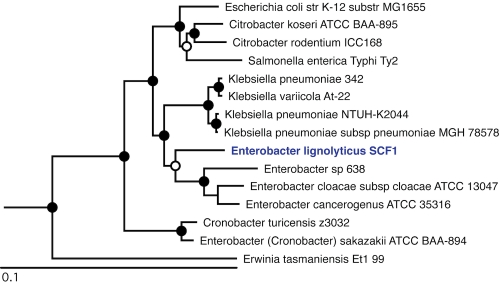
Phylogenetic tree highlighting the position of *“Enterobacter lignolyticus”* SCF1 relative to other type and non-type strains within the *Enterobacteriaceae*. Strains shown are those within the *Enterobacteriaceae* having corresponding NCBI genome project ids listed within [27]. The tree is based on a concatenated MUSCLE alignment [25] of 69 near-universal single-copy COGs (COGs 12, 13, 16, 18, 30, 41, 46, 48, 49, 52, 60, 72, 80, 81, 86, 87, 88, 89, 90, 91, 92, 93, 94, 96, 97, 98, 99, 100, 102, 103, 104, 105, 124, 126, 127, 130, 143, 149, 150, 162, 164, 172, 184, 185, 186, 197, 198, 200, 201, 202, 215, 237, 244, 256, 284, 441, 442, 452, 461, 504, 519, 522, 525, 528, 532, 533, 540, 541, 552). The tree was constructed using FastTree-2 [26] using the JTT model of amino acid evolution [28]. FastTree-2 infers approximate maximum-likelihood phylogenetic placements and provides local support values based on the Shimodaira-Hasegawa test [29]. Solid circles represent local support values over 90% and open circles over 80%. *Erwinia tasmaniensis* was used as an outgroup.

For initial genotyping and for validating the isolation, the small subunit ribosomal RNA gene was sequenced by Sanger sequencing using the universal primers 8F and 1492R [22].The 16S rRNA sequence places *“Enterobacter lignolyticus”* SCF1 in the family *Enterobacteriaceae*. However, 16S rRNA sequence is not sufficient to clearly define the evolutionary history of this region of the *Gammaproteobacteria*, and initially led to the incorrect classification of *“E. lignolyticus”* SCF1 as a member of the *Enterobacter cloacae* species. We have rectified its phylogenetic placement using the MicrobesOnline species tree [23], which is generated using 69 single-copy near-universal protein families [24] aligned by MUSCLE [25] with tree construction using FastTree-2 [26] (Figure 1).

## Genome sequencing information

### Genome project history

The genome was selected based on the ability of *“E. lignolyticus”* SCF1 to grow on and degrade lignin anaerobically. The genome sequence was completed on August 9, 2010, and presented for public access on 15 October 2010 by Genbank. Finishing was completed at Los Alamos National Laboratory. A summary of the project information is shown in Table 2, which also presents the project information and its association with MIGS version 2.0 compliance [30].

**Table 2:** Project information

**MIGS ID**	**Property**	**Term**
MIGS-31	Finishing quality	Finished
MIGS-28	Libraries used	Illumina GAii shotgun, 454 Titanium Standard, and two 454 paired-end
MIGS-29	Sequencing platforms	Illumina, 454
MIGS-31.2	Fold coverage	40× for 454 and 469× for Illumina
MIGS-30	Assemblers	Newbler, Velvet, Phrap
MIGS-32	Gene calling method	Prodigal 1.4, GenePRIMP
	Genbank ID	CP002272
	Genbank Date of Release	October 15, 2010
	GOLD ID	Gc01746
	Project relevance	Anaerobic lignin, switchgrass decomposition

### Growth conditions and DNA isolation

*“E. lignolyticus”* SCF1 grows well aerobically and anaerobically, and was routinely cultivated aerobically in 10% tryptic soy broth (TSB) with shaking at 200 rpm at 30°C. DNA for sequencing was obtained using the Qiagen Genomic-tip kit and following the manufacturer’s instructions for the 500/g size extraction. Three column preparations were necessary to obtain 50 μg of high molecular weight DNA. The quantity and quality of the extraction were checked by gel electrophoresis using JGI standards.

### Genome sequencing and assembly

The draft genome of *“Enterobacter lignolyticus”* SCF1 was generated at the DOE Joint Genome Institute (JGI) using a combination of Illumina [31] and 454 technologies [32]. For this genome we constructed and sequenced an Illumina GAii shotgun library which generated 50,578,565 reads totaling 3,844 Mb, a 454 Titanium standard library which generated 643,713 reads and two paired end 454 libraries with average insert sizes of 12517 +/- 3129 bp kb and 10286 +/- 2571 bp which generated 346,353 reads totaling 339.3 Mb of 454 data. All general aspects of library construction and sequencing performed at the JGI can be found at the JGI website [33]. The initial draft assembly contained 28 contigs in 1 scaffold. The 454 Titanium standard data and the 454 paired end data were assembled together with Newbler, version 2.3. The Newbler consensus sequences were computationally shredded into 2 kb overlapping fake reads (shreds). Illumina sequencing data was assembled with VELVET, version 0.7.63 [34], and the consensus sequences were computationally shredded into 1.5 kb overlapping fake reads (shreds). We integrated the 454 Newbler consensus shreds, the Illumina VELVET consensus shreds and the read pairs in the 454 paired end library using parallel phrap, version SPS - 4.24 (High Performance Software, LLC). The software Consed [35-37] was used in the following finishing process. Illumina data was used to correct potential base errors and increase consensus quality using the software Polisher developed at JGI (Alla Lapidus, unpublished). Possible mis-assemblies were corrected using gapResolution (Cliff Han, unpublished), Dupfinisher [38], or sequencing cloned bridging PCR fragments with subcloning. Gaps between contigs were closed by editing in Consed, by PCR and by Bubble PCR (J-F Cheng, unpublished) primer walks. A total of 198 additional reactions were necessary to close gaps and to raise the quality of the finished sequence. The total size of the genome is 4,814,049 bp and the final assembly is based on 191.3 Mb of 454 draft data, which provided an average 40× coverage of the genome, and 2249.8 Mb of Illumina draft data, which provided an average 469× coverage of the genome; the coverage from different technologies is reported separately because they have different error patterns.

### Genome annotation

Protein coding genes were identified using Prodigal [39] and tRNA, rRNA and other RNA genes using tRNAscan-SE [40], RNAmmer [41] and Rfam [42] as part of the ORNL genome annotation pipeline followed by a round of manual curation using the JGI GenePRIMP pipeline [43]. The predicted CDSs were translated and used to search the National Center for Biotechnology Information (NCBI) nonredundant database, UniProt, TIGR-Fam, Pfam, PRIAM, KEGG, COG, and InterPro databases. Additional gene prediction analysis and functional annotation were performed within the Integrated Microbial Genomes - Expert Review (IMG-ER) platform [44] using the JGI standard annotation pipeline [45,46].

## Genome properties

**Figure 2:**
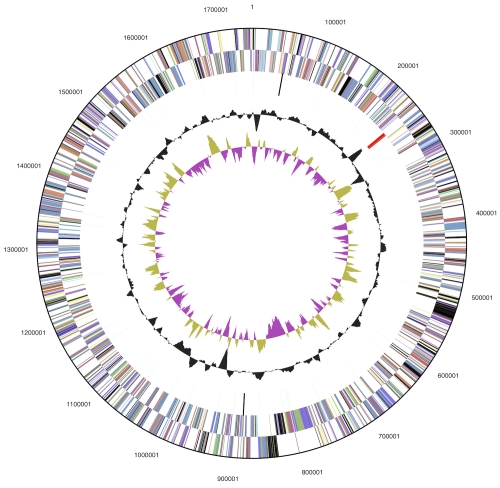
Graphical circular map of the genome. From outside to the center: Genes on forward strand (color by COG categories), Genes on reverse strand (color by COG categories), RNA genes (tRNAs green, rRNAs red, other RNAs black), GC content, GC skew.

The genome consists of a 4,814,049 bp circular chromosome with a GC content of 57.02% (Table 3 and Figure 2). Of the 4,556 genes predicted, 4,449 were protein-coding genes, and 107 RNAs; 50 pseudogenes were also identified. The majority of the protein-coding genes (85.8%) were assigned with a putative function while the remaining ones were annotated as hypothetical proteins. The distribution of genes into COGs functional categories is presented in Table 4, Table5 and Table 6.

**Table 3:** Nucleotide content and gene count levels of the genome

**Attribute**	**Value**	**% of Total**
Genome size (bp)	4,814,049	100.00%
DNA coding region (bp)	4,312,328	89.58%
DNA G+C content (bp)	2,744,879	57.02%
Number of replicons	1	
Extrachromosomal elements	0	
Total genes	4,556	100.00%
RNA genes	107	2.35%
rRNA operons	7	
Protein-coding genes	4,449	97.65%
Pseudo genes	50	1.10%
Genes with function prediction	3,909	85.80%
Genes in paralog clusters	823	18.06%
Genes assigned to COGs	3,743	82.16%
Genes assigned Pfam domains	3,995	87.69%
Genes with signal peptides	1,009	22.15%
Genes with transmembrane helices	1,108	24.32%
CRISPR-associated genes (CAS)	0	% of Total

**Table 4:** Number of genes associated with the 25 general COG functional categories

**Code**	**Value**	**%age**^a^	**Description**
J	184	4.37	Translation
A	1	0.02	RNA processing and modification
K	360	8.54	Transcription
L	155	3.68	Replication, recombination and repair
B	0	0	Chromatin structure and dynamics
D	33	0.78	Cell cycle control, mitosis and meiosis
Y	0	0	Nuclear structure
V	48	1.14	Defense mechanisms
T	219	5.20	Signal transduction mechanisms
M	239	5.67	Cell wall/membrane biogenesis
N	138	3.27	Cell motility
Z	0	0	Cytoskeleton
W	1	0.02	Extracellular structures
U	150	3.56	Intracellular trafficking and secretion
O	140	3.32	Posttranslational modification, protein turnover, chaperones
C	275	6.52	Energy production and conversion
G	432	10.25	Carbohydrate transport and metabolism
E	415	9.85	Amino acid transport and metabolism
F	98	2.33	Nucleotide transport and metabolism
H	176	4.18	Coenzyme transport and metabolism
I	108	2.56	Lipid transport and metabolism
P	235	5.58	Inorganic ion transport and metabolism
Q	85	2.02	Secondary metabolites biosynthesis, transport and catabolism
R	409	9.70	General function prediction only
S	314	7.45	Function unknown
-	813	17.84	Not in COGs

a) The total is based on the total number of protein coding genes in the annotated genome.

**Table 5:** Number of non-orthologous protein-coding genes found in *“Enterobacter lignolyticus”* SCF1 with respect to related genomes

**Species**	**Number of distinct genes in** ***“E. lignolyticus”* SCF1**
*Enterobacter* sp. 638	1,580
*Enterobacter cancerogenus* ATCC 35316	1,551*
*Enterobacter cloacae* ATCC 13047	2,891*
*Klebsiella pneumoniae* 342	1,389
*Klebsiella pneumoniae* MGH 78578	1,451
*Klebsiella pneumoniae* NTUH-K2044	1,424
*Klebsiella variicola* At-22	1,394
*Citrobacter koseri* ATCC BAA-895	1,507
*Citrobacter rodentium* ICC168	1,682
*Escherichia coli* K-12 MG1655	1,654
*Salmonella enterica* Typhi Ty2	1,811
*Cronobacter turicensis* z3032	1,875
*Cronobactersakazakii* ATCC BAA-894	1,918
*Erwinia tasmaniensis* Et1/99	2,392
Protein-coding genes distinct in *“E. lignolyticus”* SCF1 compared with all orthologous genes found in above genomes	643

* Based on incompletely annotated genome.

**Table 6:** Number of genes not found in near-relatives associated with the 25 general COG functional categories*

**Code**	**Value**	**Description**
-	151	Hypothetical (no conserved gene family)
-	17	Transposase / Integrase (annotation-based)
-	80	Transport (annotation-based)
-	66	Signaling and Regulation
J	6	Translation
A	0	RNA processing and modification
K	51	Transcription
L	18	Replication, recombination and repair
B	0	Chromatin structure and dynamics
D	2	Cell cycle control, mitosis and meiosis
Y	0	Nuclear structure
V	7	Defense mechanisms
T	30	Signal transduction mechanisms
M	41	Cell wall/membrane biogenesis
N	20	Cell motility
Z	0	Cytoskeleton
W	1	Extracellular structures
U	22	Intracellular trafficking and secretion
O	9	Posttranslational modification, protein turnover, chaperones
C	20	Energy production and conversion
G	68	Carbohydrate transport and metabolism
E	28	Amino acid transport and metabolism
F	5	Nucleotide transport and metabolism
H	5	Coenzyme transport and metabolism
I	14	Lipid transport and metabolism
P	23	Inorganic ion transport and metabolism
Q	8	Secondary metabolites biosynthesis, transport and catabolism
R	43	General function prediction only
S	23	Function unknown
-	255	Not in COGs

* Number of genes from set of 643 genes not found in near-relatives associated with the 25 general COG functional categories and several annotation-based classifications. Note that counts do not sum to 643 genes as a given gene is sometimes classified in more than one COG functional category.

## Lignocellulose degradation pathways

*“E. lignolyticus”* SCF1 has a relatively small arsenal of lignocellulolytic carbohydrate active enzymes, including a single GH8 endoglucanase, and a GH3 beta-glucosidase, but no xylanase or beta-xylosidase. Table 7 provides a more complete list of lignocellulolytic enzymes. The genome also contains a large number of saccharide and oligosaccharide transporters, including several ribose ABC transporters, a xylose ABC transporter (Entcl_0174-0176), and multiple cellobiose PTS transporters (Entcl_1280, Entcl_2546-2548, Entcl_3764, Entcl_4171-4172).

**Table 7:** Selection of lignocellulolytic carbohydrate active, lignin oxidative (LO) and lignin degrading auxiliary (LDA) enzymes [47,48]†.

**Locus Tag**	**Family**	**Function**
Entcl_0212	GH8	endoglucanase (EC 3.2.1.4)
Entcl_1570	GH3	beta-glucosidase (EC 3.2.1.21)
Entcl_0851	GH1	6-phospho-beta-glucosidase (EC 3.2.1.86)
Entcl_0991	GH1	6-phospho-beta-glucosidase (EC 3.2.1.86)
Entcl_1274	GH1	6-phospho-beta-glucosidase (EC 3.2.1.86)
Entcl_3004	GH1	6-phospho-beta-glucosidase (EC 3.2.1.86)
Entcl_3339	GH2	beta-galactosidase (EC 3.2.1.23)
Entcl_0624	GH2	beta-galactosidase (EC 3.2.1.23)
Entcl_2579	GH2	beta-mannosidase (EC 3.2.1.25)
Entcl_2687	GH3	beta-N-acetylhexosaminidase (EC 3.2.1.52)
Entcl_3271	GH4	alpha-galactosidase (EC 3.2.1.22)
Entcl_0170	GH13	alpha-amylase (EC 3.2.1.1)
Entcl_3416	GH13	alpha-glucosidase (EC 3.2.1.20)
Entcl_2926	GH18	chitinase (EC 3.2.1.14)
Entcl_2924	GH19	chitinase (EC 3.2.1.14)
Entcl_4037	GH35	beta-galactosidase (EC 3.2.1.23)
Entcl_3090	GH38	alpha-mannosidase (EC 3.2.1.24)
Entcl_0250	CE4	polysaccharide deacetylase (EC 3.5.-.-)
Entcl_3596	CE4	polysaccharide deacetylase (EC 3.5.-.-)
Entcl_3059	CE8	pectinesterase (EC 3.1.1.11)
Entcl_2112	LDA2	vanillyl-alcohol oxidase (EC 1.1.3.38)
Entcl_1569	LDA2	D-lactate dehydrogenase (EC 1.1.1.28)
Entcl_4187	LDA2	UDP-N-acetylmuramate dehydrogenase (EC 1.1.1.158)
Entcl_3603	LO1	putative laccase (EC 1.10.3.2)
Entcl_0735	LO1	putative laccase (EC 1.10.3.2)
Entcl_4301	LO2	catalase/peroxidase (EC 1.11.1.6, 1.11.1.7)

† Enzyme families are as per the CAZy and FOLy databases

**Figure 3:**
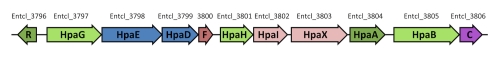
The entire 4-hydroxyphenylacetate degradation pathway is encoded in a single gene cluster HpaRGEDFHIXABC, including a divergently expressed regulator (HpaR), and a 4-hydroxyphenylacetate permease (HpaX).

**Figure 4:**
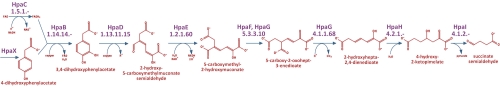
The 4-hydroxyphenylacetate degradation pathway via homoprotocatechuate (3,4-dihydroxyphenylacetate).

The mechanisms for lignin degradation in bacteria are still poorly understood. Two multi-copper oxidases (putative laccases) and a putative peroxidase (see Table 7) may be involved in oxidative lignin degradation. We also found multiple glutathione S-transferase proteins, and it is possible that one or more of these may be involved in cleavage of beta-aryl ether linkages, as is the case with LigE/LigF in *Sphingomonas paucimobilis* [49]. However, *“E. lignolyticus”* SCF1 does not seem to posses the core protocatechuate and 3-O-methylgallate degradation pathways responsible for lignin catabolism in *S. paucimobilis*. Instead, lignin catabolism may proceed via homoprotocatechuate through the 4-hydroxyphenylacetate degradation pathway, encoded on a gene cluster conserved between other *Enterobacter, Klebsiella*, and some *E. coli* strains (Figures 3, 4).

### Lignin degradation

**Figure 5:**
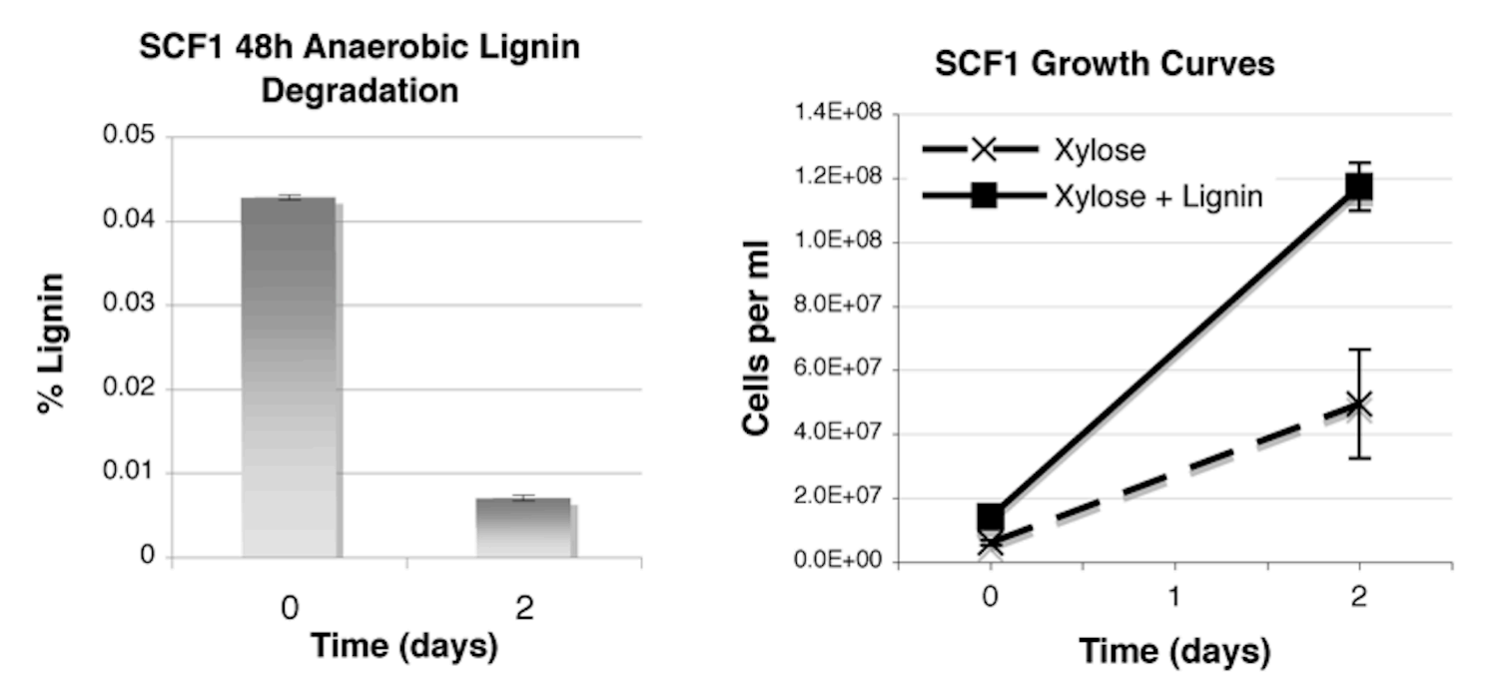
Anaerobic lignin degradation by *“E. lignolyticus”* SCF1 after 48 hours in culture, grown with xylose minimal media.

We have grown SCF1 in xylose minimal media with and without lignin, and measured both cell counts (by acridine orange direct counts) and lignin degradation (by change in absorbance at 280 nm) over time. Lignin degradation was substantial after two days (left), and significantly enhanced growth of cells in culture (right); data are expressed as mean with standard deviation (n=3, Figure 5). Further studies will explore the moieties of lignin used in anaerobic growth as well as explore growth on and utilization of other types of lignin.

### Phenotypic Microarray

We used the Biolog phenotypic microarray to test the range of growth conditions. For each of the eight plates in the array, *“E. lignolyticus”* SCF1 cells were grown up on 10% TSB agar plates, scraped off and resuspended in 20mM D-Glucose MOD-CCMA, adjusted to 0.187 OD, 1× concentrate of Biolog Dye Mix G added, and then inoculated. PM plates include two plates with different carbon sources (PM 1 and 2a), one plate of different simple nitrogen sources (PM 3b), one plates of phosphorous and sulfur sources (PM4A), one plate of nutritional supplements (PM5), and three plates of amino acid dipeptides as nitrogen sources (PM6, PM7, PM8). Carbon source, D-Glucose, was omitted from MOD-CCMA when used to inoculate PM1 and 2a. Similarly, NH_4_Cl, KH_2_PO_4_ and vitamins were omitted from 20mM D-Glucose MOD CCMA when inoculating plates containing nitrogen sources, phosphorus/sulfur sources, and nutrient supplements, respectively. On plates 6-8, the positive control is L-Glutamine. The phenotypic microarray revealed a number of carbon and nitrogen sources that resulted in four times the growth or more compared to the negative control based on duplicate runs (Table 8 and 9), as well as sulfur and phosphorous sources that improved growth by 10% or more (Tables 10 and 11). None of the dipeptides resulted in an increase in growth more than twice the background, and so are not reported here. Of the nutritional supplements tested in PM5, 2'-deoxyuridine and 2'-deoxyadenosine resulted in 10% growth improvement, while (5) 4-amino-imidazole-4(5)-carboxamide, Tween 20, Tween 40, Tween 60, and Tween 80 resulted in 20% growth improvement.

**Table 8:** Carbon source by phenotypic array (PM 1 and 2a)

**Chemical Name**	**KEGG**	**CAS**	**Ratio to background**
D-Fructose	C00095	57-48-7	8.48
D-Sorbitol	C00794	50-70-4	8.36
N-Acetyl-D-Glucosamine	C03000	7512-17-6	8.30
D-Gluconic Acid	C00257	527-07-1	8.28
D-Trehalose	C01083	99-20-7	8.18
D-Mannose	C00159	3458-28-4	8.10
D-Xylose	C00181	58-86-6	8.09
a-D-Glucose	C00031	50-99-7	8.07
N-Acetyl-D-Mannosamine	C00645	7772-94-3	7.92
D-Mannitol	C00392	69-65-8	7.92
D-Galactose	C00124	59-23-4	7.92
D-Glucosaminic Acid	C03752	3646-68-2	7.85
D-Ribose	C00121	50-69-1	7.76
b-Methyl-D-Glucoside		709-50-2	7.70
D-Glucuronic Acid	C00191	14984-34-0	7.69
D-Glucosamine	C00329	66-84-2	7.68
D-Galactonic Acid-g-Lactone	C03383	2782-07-2	7.67
Maltose	C00208	69-79-4	7.62
2-Deoxy-D-Ribose	C01801	533-67-5	7.57
Glycerol	C00116	56-81-5	7.52
m-Hydroxyphenyl Acetic Acid	C05593	621-37-4	7.42
L-Arabinose	C00259	87-72-9	7.40
m-Inositol	C00137	87-89-8	7.39
L-Serine	C00065	56-45-1	7.38
3-Methylglucose		13224-94-7	7.36
Maltotriose	C01835	1109-28-0	7.30
D-Melibiose	C05402	585-99-9	7.25
L-Fucose	C01019	2438-80-4	7.25
D-Arabinose	C00216	10323-20-3	7.10
Hydroxy-L-Proline	C01015	51-35-4	7.08
2'-Deoxyadenosine	C00558	16373-93-6	7.02
L-Alanine	C00041	56-41-7	6.94
Tyramine	C00483	60-19-5	6.93
Gly-Pro		704-15-4	6.93
D-Galacturonic Acid	C00333	91510-62-2	6.91
L-Rhamnose	C00507	3615-41-6	6.86
p-Hydroxyphenyl Acetic Acid	C00642	156-38-7	6.83
Acetic Acid	C00033	127-09-3	6.81
L-Proline	C00148	147-85-3	6.80
Fumaric Acid	C00122	17013-01-3	6.80
D,L-Malic Acid	C00497	6915-15-7	6.75
D,L-Lactic acid	C01432	312-85-6	6.71
Dihydroxyacetone	C00184	96-26-4	6.69
Tween 20	C11624	9005-64-5	6.57
N-Acetyl-D-Galactosamine		14215-68-0	6.45
Inosine	C00294	58-63-9	6.45
Ala-Gly		687-69-4	6.43
L-Histidine	C00135	5934-29-2	6.37
D-Alanine	C00133	338-69-2	6.29
D-Fructose-6-Phosphate	C00085	26177-86-637250-85-4	6.25
L-Glutamine	C00064	56-85-9	6.08
Gly-Glu		7412-78-4	6.00
D-Cellobiose	C00185	528-50-7	5.98
D-Glucose-1-Phosphate	C00103	56401-20-8	5.95
D-Psicose	C06468	551-68-8	5.92
Citric Acid	C00158	6132-04-3	5.91
L-Glutamic Acid	C00025	6106-04-3	5.84
b-Methyl-D-Galactoside	C03619	1824-94-8	5.70
L-Aspartic Acid	C00049	3792-50-5	5.65
D-Serine	C00740	312-84-5	5.63
Methylpyruvate		600-22-6	5.62
Pyruvic Acid	C00022	113-24-6	5.56
Propionic Acid	C00163	137-40-6	5.48
Melibionic Acid		70803-54-2	5.43
D-Malic Acid	C00497	636-61-3	5.38
D-Aspartic Acid	C00402	1783-96-6	5.38
5-Keto-D-Gluconic Acid	C01062	91446-96-7	5.37
Succinic Acid	C00042	6106-21-4	5.35
Gly-Asp	C02871		5.28
D,L-a-Glycerol Phosphate	C00093	3325-00-6	5.26
Putrescine	C00134	333-93-7	5.14
Gentiobiose	C08240	554-91-6	5.00
D-Glucose-6-Phosphate	C00092	3671-99-6	4.90
a-Methyl-D-Galactoside	C03619	3396-99-4	4.84
Uridine	C00299	58-96-8	4.68
Bromosuccinic Acid		923-06-8	4.68
Thymidine	C00214	50-89-5	4.63
L-Asparagine	C00152	70-47-3	4.55
a-Hydroxybutyric Acid	C05984	19054-57-0	4.38
L-Malic Acid	C00149	138-09-0	4.34
L-Ornithine	C00077	3184-13-2	4.28
N-Acetyl-D-glucosaminitol		4271-28-7	4.23
L-Lyxose	C01508	1949-78-6	4.23
L-Threonine	C00188	72-19-5	4.21
g-Amino-N-Butyric Acid	C00334	56-12-2	4.19
Arbutin	C06186	497-76-7	4.17

**Table 9:** Nitrogen sources by phenotypic array (PM 3b)

**Chemical Name**	**KEGG**	**CAS**	**Ratio to background**
Gly-Gln		13115-71-4	5.63
Gly-Asn			5.63
L-Cysteine	C00097	7048-04-6	5.29
Gly-Glu		7412-78-4	5.26
Ala-Gln		39537-23-0	4.92
Ala-Asp	C02871	20727-65-5	4.58
L-Aspartic Acid	C00049	3792-50-5	4.33
L-Glutamine	C00064	56-85-9	4.03

**Table 10:** Phosphorous source by phenotypic array (PM 4a)

Chemical Name	KEGG	CAS	Ratio to background
O-Phospho-D-Serine		73913-63-0	1.42
Phospho-Glycolic Acid	C00988		1.28
Carbamyl Phosphate	C00416	72461-86-0	1.26
O-Phospho-L-Threonine		1114-81-4	1.25
Tripolyphosphate	C02466		1.24
O-Phospho-L-Serine		407-41-0	1.23
Cysteamine-S-Phosphate		3724-89-8	1.22
Cytidine 2'-Monophosphate	C03104	85-94-9	1.21
Guanosine 5'-Monophosphate	C00144	5550-12-9	1.21
Guanosine 3'-Monophosphate	C06193		1.20
Phosphoenol Pyruvate	C00074	5541-93-5	1.20
Cytidine 3'-Monophosphate	C05822	84-52-6	1.20
Cytidine 5'-Monophosphate	C00055	6757-06-8	1.20
Adenosine 2',3'-Cyclic Monophosphate		37063-35-7	1.20
Phospho-L-Arginine		108321-86-4	1.20
Adenosine 3'-Monophosphate	C01367	84-21-9	1.20
Guanosine 2',3'-Cyclic Monophosphate		15718-49-7	1.19
D-3-Phospho-Glyceric Acid	C00631	80731-10-8	1.19
Phosphate	C00009	10049-21-5	1.19
Guanosine 2'-Monophosphate		6027-83-4	1.19
Thiophosphate		10489-48-2	1.18
Thymidine 3'-Monophosphate		108320-91-8	1.18
Thymidine 5'-Monophosphate	C00364	33430-62-5	1.16
6-Phospho-Gluconic Acid		53411-70-4	1.16
Dithiophosphate			1.16
2-Aminoethyl Phosphonic Acid	C03557	2041-14-7	1.15
Phosphoryl Choline	C00588	4826-71-5	1.14
D,L-a-Glycerol Phosphate	C00093	3325-00-6	1.13
Trimetaphosphate	C02466	7785-84-4	1.13

**Table 11:** Sulfur source by phenotypic array (PM 4a)

Chemical Name	KEGG	CAS	Ratio to background
L-Cysteine Sulfinic Acid	C00607	1115-65-7	1.24
Gly-Met		554-94-9	1.23
Tetramethylene Sulfone		126-33-0	1.21
L-Methionine	C00073	63-68-3	1.21
N-Acetyl-D,L-Methionine	C02712	71463-44-0	1.20
L-Methionine Sulfoxide	C02989	3226-65-1	1.19
Tetrathionate	C02084	13721-29-4	1.18
L-Cysteine	C00097	7048-04-6	1.17
Sulfate	C00059	7727-73-3	1.14
L-Djenkolic Acid	C08275	28052-93-9	1.14
Cys-Gly		19246-18-5	1.13

## Conclusion

Close relatives of *“Enterobacter lignolyticus”* SCF1 were isolated seven independent times from Puerto Rico tropical forest soils, growing anaerobically with lignin or switchgrass as the sole carbon source, suggesting that it is relatively abundant in tropical forest soils and has broad capability for deconstruction of complex heteropolymers such as biofuel feedstocks. In a previous study, *Enterobacter* was one of four isolates from the poplar rhizosphere chosen for genomic sequencing because of its ability to improve the carbon sequestration ability of poplar trees when grown in poor soils [50].

Isolates from the *Enterobacteriaceae* are extremely genetically diverse despite the near identity of genotypic markers such as small subunit ribosomal (16S) RNA genes. Multi-locus sequence typing and comparative genomic hybridization show that the isolates seem to fall into two distinct clades: the first being more homogeneous and containing isolates found in hospitals, and the second being more diverse and found in a broader array of environments [51].

This organism was determined to grow aerobically and anaerobically, and when screening for enzyme activity, the enzymes isolated showed accelerated phenol oxidase and peroxidase enzyme activity under aerobic conditions. In addition, this organism is capable of growth in 8% ethyl-methylimidazolium chloride ([C_2_mim]Cl), an ionic liquid being studied for pre-treatment of feedstocks. This extremely high tolerance to ionic liquids is potentially quite useful for industrial biofuels production from feedstocks and the mechanism is currently under investigation.
